# Crystal structure of 4-(6-chloro-4-oxo-4*H*-chromen-3-yl)-2-methyl­amino-3-nitro-4*H*,5*H*-pyrano[3,2-*c*]chromen-5-one chloro­form monosolvate

**DOI:** 10.1107/S2056989015011810

**Published:** 2015-06-27

**Authors:** Rajamani Raja, Subramani Kandhasamy, Paramasivam T. Perumal, A. SubbiahPandi

**Affiliations:** a Department of Physics, Presidency College (Autonomous), Chennai 600 005, India; b Organic Chemistry Division, CSIR Central Leather Research Institute, Chennai 600 020, India

**Keywords:** crystal structure, chromene, chromones, pyran, hydrogen bonding, C—H⋯π inter­actions, π–π inter­actions, inversion dimers

## Abstract

In the title compound, C_23_H_14_Cl_4_N_2_O_7_, the pyran ring has an envelope conformation with the methine C atom as the flap. The chromene rings are almost planar (r.m.s. deviations of 0.027 and 0.018 Å) and their mean planes are inclined to one another by 85.61 (10)°. The mean planes of the pyran ring and the chromene ring fused to it are inclined to one another by 7.41 (13)°. The mol­ecular structure is stabilized by an intra­molecular N—H⋯O hydrogen bond, generating an *S*(6) ring motif. In the crystal, mol­ecules are linked by pairs of N—H⋯O hydrogen bonds, forming inversion dimers with an *R* ^2^_2_(12) ring motif. The dimers are linked by pairs of C—H⋯O hydrogen bonds, enclosing *R* ^2^_2_(18) ring motifs, forming chains along [010]. Within the chains there are C—H⋯π inter­actions. The chains are linked *via* slipped parallel π–π inter­actions, forming a three-dimensional structure [the shortest inter-centroid distance is 3.7229 (19) Å].

## Related literature   

For the uses and biological importance of chromones, see: Miao & Yang (2000 ▸); Lin *et al.* (2000 ▸); Larget *et al.* (2000 ▸); Groweiss *et al.* (2000 ▸); Deng *et al.* (2000 ▸); Pietta (2000 ▸); Mori *et al.* (1998 ▸); Montaña *et al.* (2007 ▸); Hsu *et al.* (2006 ▸); Beecher (2003 ▸). For a related structure, see: Narayanan *et al.* (2013 ▸).
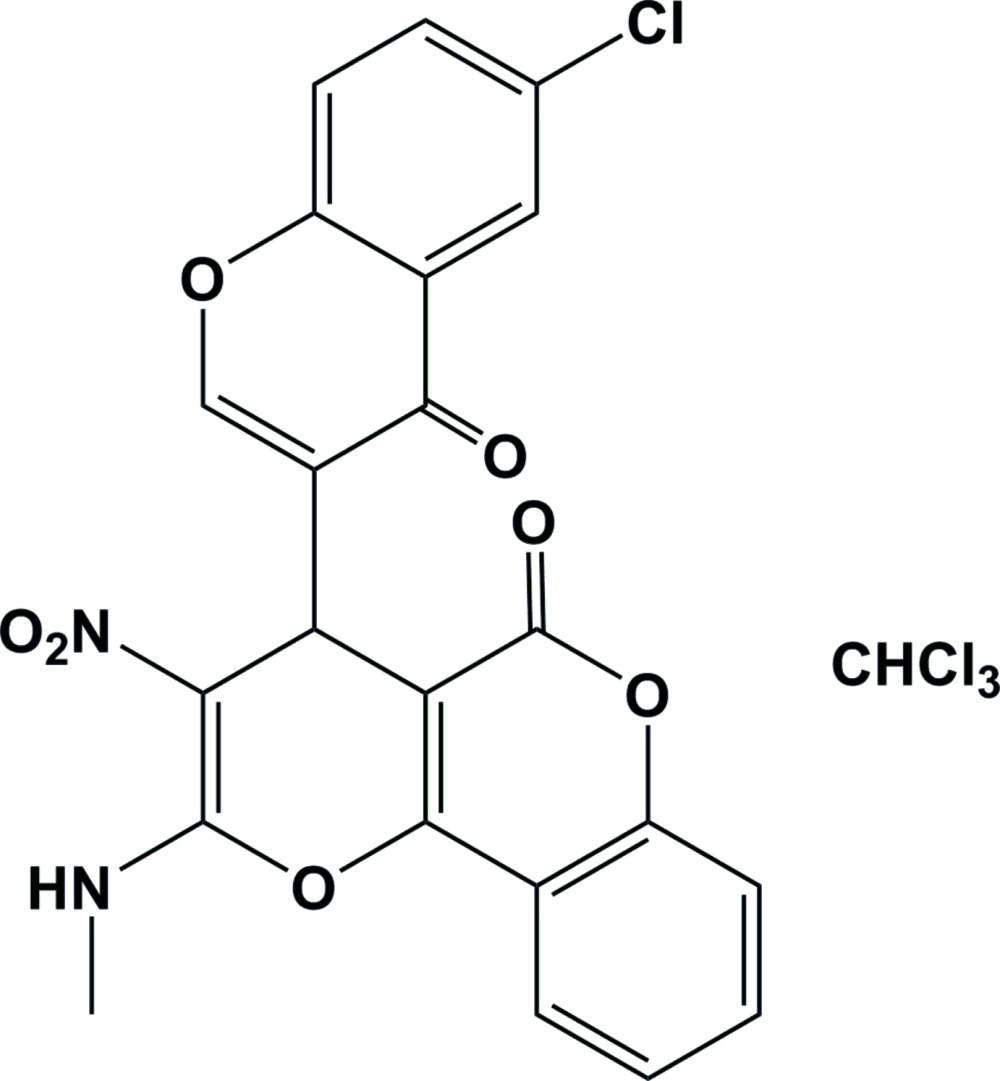


## Experimental   

### Crystal data  

C_22_H_13_ClN_2_O_7_·CHCl_3_
*M* *_r_* = 572.16
Triclinic, 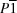

*a* = 8.3716 (2) Å
*b* = 11.6435 (3) Å
*c* = 13.1018 (4) Å
α = 86.455 (1)°
β = 88.251 (1)°
γ = 69.841 (1)°
*V* = 1196.51 (6) Å^3^
*Z* = 2
Mo *K*α radiation
μ = 0.54 mm^−1^
*T* = 293 K
0.25 × 0.20 × 0.20 mm

### Data collection  

Bruker SMART APEXII CCD diffractometer
Absorption correction: multi-scan (*SADABS*; Bruker, 2008 ▸) *T*_min_ = 0.878, *T*_max_ = 0.897
15560 measured reflections
4208 independent reflections
3451 reflections with *I* > 2σ(*I*)
*R*_int_ = 0.019

### Refinement  

*R*[*F* ^2^ > 2σ(*F* ^2^)] = 0.055
*wR*(*F* ^2^) = 0.154
*S* = 1.03
4208 reflections
325 parameters
H-atom parameters constrained
Δρ_max_ = 0.73 e Å^−3^
Δρ_min_ = −0.89 e Å^−3^

### 

Data collection: *APEX2* (Bruker, 2008 ▸); cell refinement: *SAINT* (Bruker, 2008 ▸); data reduction: *SAINT*; program(s) used to solve structure: *SHELXS97* (Sheldrick, 2008 ▸); program(s) used to refine structure: *SHELXL97* (Sheldrick, 2008 ▸); molecular graphics: *PLATON* (Spek, 2009 ▸); software used to prepare material for publication: *SHELXL97* and *PLATON* (Spek, 2009 ▸).

## Supplementary Material

Crystal structure: contains datablock(s) global, I. DOI: 10.1107/S2056989015011810/su5158sup1.cif

Structure factors: contains datablock(s) I. DOI: 10.1107/S2056989015011810/su5158Isup2.hkl

Supporting information file. DOI: 10.1107/S2056989015011810/su5158Isup3.cml

**Fig. 1.**
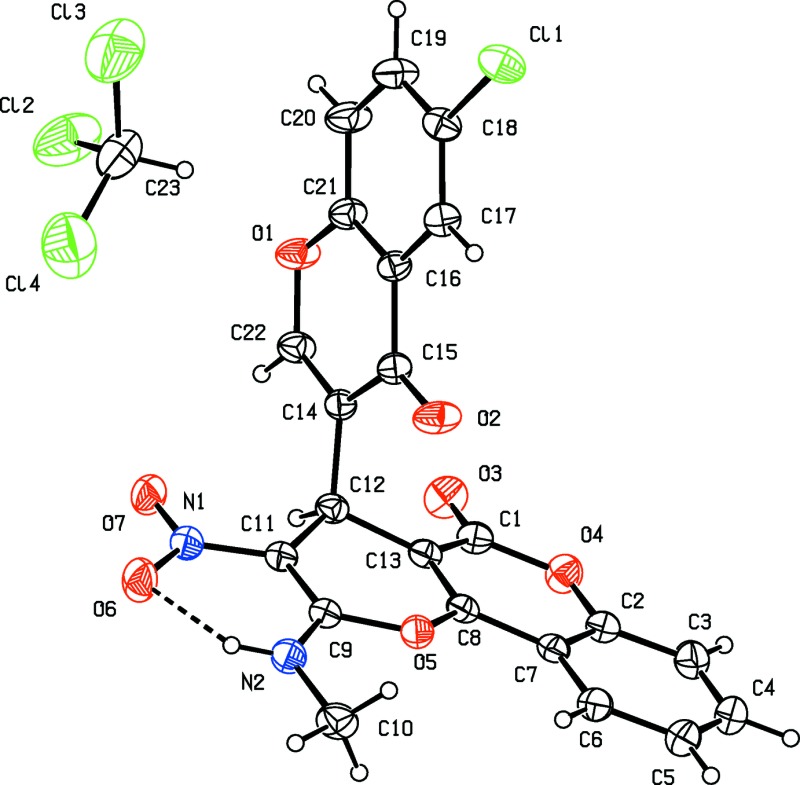
The molecular structure of the title compound, with atom labelling. Displacement ellipsoids are drawn at the 30% probability level. The intramolecular N—H···O hydrogen bond is shown as a dashed line (see Table 1 for details)

**Fig. 2.**
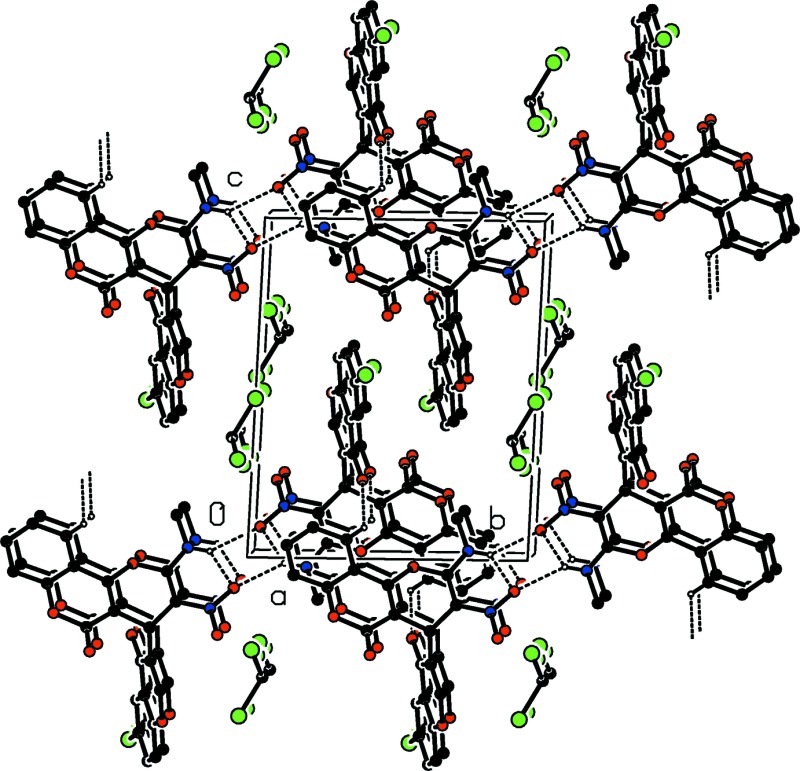
The crystal packing of the title compound, viewed along the a axis. The hydrogen bonds are shown as dashed lines (see Table 1 for details). H atoms not involved in these interactions have been omitted for clarity.

. DOI: 10.1107/S2056989015011810/su5158fig1.tif
The mol­ecular structure of the title compound, with atom labelling. Displacement ellipsoids are drawn at the 30% probability level. The intra­molecular N—H⋯O hydrogen bond is shown as a dashed line (see Table 1 for details)

a . DOI: 10.1107/S2056989015011810/su5158fig2.tif
The crystal packing of the title compound, viewed along the *a* axis. The hydrogen bonds are shown as dashed lines (see Table 1 for details). H atoms not involved in these inter­actions have been omitted for clarity.

CCDC reference: 1055442

Additional supporting information:  crystallographic information; 3D view; checkCIF report

## Figures and Tables

**Table 1:** Hydrogen-bond geometry (, ) *Cg*4 is the centroid of the C2C7 ring.

*D*H*A*	*D*H	H*A*	*D* *A*	*D*H*A*
N2H2O6	0.86	1.00	2.604(3)	127
N2H2O6^i^	0.86	2.17	2.910(3)	144
C6H6O2^ii^	0.96	2.49	3.186(4)	132
C10H10*B* *Cg*4^ii^	0.98	2.98	3.719(4)	134

Symmetry codes: (i) 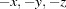; (ii) 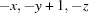.

## supplementary crystallographic information

### S1. Structural commentary

\ Chromones constitute a major class of naturally occurring compounds and inter­est in their chemistry continues unabated because of their usefulness as biologically active agents (Miao & Yang, 2000). Some of the biological activities attributed to chromone derivatives include cytotoxic anti­cancer (Lim *et al.*, 2000), neuroprotective (Larget *et al.*, 2000), HIV-inhibitory (Groweiss *et al.*, 2000), anti­microbial (Deng *et al.*, 2000), anti­fungal (Mori *et al.*, 1998) and anti­oxidant activities (Pietta, 2000). Chromone derivatives are present in large amounts in the human diet (Beecher, 2003), due to their abundance in plants and their low mammalian toxicity. They are known to exhibit anti­oxidant (Montaña *et al.*, 2007), anti-inflammatory, anti­microbial, anti­hypertensive, anti­platelet, gastroprotective, anti­tumour (Hsu *et al.*, 2006) and anti­allergic activities.

The molecular structure of the title compound is illustrated in Fig. 1. It exhibits structural similarities with a related chromenone derivative, 4-(4-Bromo­phenyl)-2-methyl­amino-3-nitro-5,6,7,8-tetra­hydro-4*H*-chromen-\ 5-one (Narayanan *et al.*, 2013). The chromene rings (A = O4/C1—C8/C13) and (B = O1/C14—C22) are almost planar (r.m.s. deviations of 0.027 and 0.018 Å, respectively) and normal to one another with a dihedral angle of 85.61 (10) ° between their mean planes. The pyran ring (C = O5/C8/C9/C11—C13) has an envelope conformation with atom C12 as the flap. Its mean plane is inclined to the mean plane of the chromene ring A, to which it is fused, by 7.41 (13) °. The nitro group is almost coplanar to the pyran ring, as indicated by torsion angles C12—C11—N1—O7 = 1.2 (4) \% and C9—C11—N1—O6 = 2.5 (5) °. The molecular structure is stabilized by an intra­molecular N—H···O hydrogen bond, which generates an S(6) ring motif (Table 1 and Fig. 1).

In the crystal, molecules are linked by a pair of N—H···O hydrogen bonds forming inversion dimers with an R^2^_2_(12) ring motif; Table 1 and Fig. 2. The dimers are linked by a pair of C—H···O hydrogen bonds, enclosing R^2^_2_((18) ring motifs, and forming chains along [010]. Within the chains there are C—H···π inter­actions (Table 1). The chains are linked via slipped parallel π-π inter­actions forming a three-dimensional structure [Cg2···Cg2^i^ = 3.9337 (16) Å, inter-planar distance = 3.5746 (12) Å, slippage 1.642 Å; Cg5···Cg5^ii^ = 3.7229 (19) Å, inter-planar distance = 3.4023 (14) Å, slippage = 1.511 Å; symmetry codes: (i) -x-1, -y+1, -z; (ii) -x, -y+1, -z+1].

### S2. Synthesis and crystallization

A three component coupling reaction, involving 4-hy­droxy­coumarin (0.81 g, 5 mmol), 6-chloro-4-oxo-4*H*-chromene-3-carbaldehyde (0.87 g, 5 mmol) and NMSM (0.74 g, 5 mmol), was carried out in EtOH at room temperature (3 h) in the presence of tri­ethyl­amine (0.1eq) as catalyst. Upon completion of the reaction, the mixture was filtered, and washed with ethanol to obtain the desired product as a white solid. Using this combination of ethanol and tri­ethyl­amine gave an excellent result with a shorter than normal reaction time and an overall yield of 83 %. The title compound was recrystallized from chloro­form giving colourless block-like crystals.

### S3. Refinement

Crystal data, data collection and structure refinement details are summarized in Table 2. The N and C-bound H atoms were positioned geometrically and allowed to ride on their parent atoms: N—H = 0.86 Å, C–H = 0.93–0.98 Å with *U*_iso_(H) = 1.5*U*_eq_(C) for methyl H atoms and 1.2*U*_eq_(N,C) for all other H atoms.

### Crystal data

**Table:**

C_22_H_13_ClN_2_O_7_·CHCl_3_	*Z* = 2
*M**_r_* = 572.16	*F*(000) = 580
Triclinic, *P*1	*D*_x_ = 1.588 Mg m^−^^3^
Hall symbol: -P 1	Mo *K*α radiation, λ = 0.71073 Å
*a* = 8.3716 (2) Å	Cell parameters from 3451 reflections
*b* = 11.6435 (3) Å	θ = 1.6–25.0°
*c* = 13.1018 (4) Å	µ = 0.54 mm^−^^1^
α = 86.455 (1)°	*T* = 293 K
β = 88.251 (1)°	Block, colourless
γ = 69.841 (1)°	0.25 × 0.20 × 0.20 mm
*V* = 1196.51 (6) Å^3^	

### Data collection

**Table:**

Bruker SMART APEXII CCD diffractometer	4208 independent reflections
Radiation source: fine-focus sealed tube	3451 reflections with *I* > 2σ(*I*)
Graphite monochromator	*R*_int_ = 0.019
ω and φ scans	θ_max_ = 25.0°, θ_min_ = 1.6°
Absorption correction: multi-scan (*SADABS*; Bruker, 2008)	*h* = −9→9
*T*_min_ = 0.878, *T*_max_ = 0.897	*k* = −13→13
15560 measured reflections	*l* = −15→15

### Refinement

**Table:**

Refinement on *F*^2^	Primary atom site location: structure-invariant direct methods
Least-squares matrix: full	Secondary atom site location: difference Fourier map
*R*[*F*^2^ > 2σ(*F*^2^)] = 0.055	Hydrogen site location: inferred from neighbouring sites
*wR*(*F*^2^) = 0.154	H-atom parameters constrained
*S* = 1.03	*w* = 1/[σ^2^(*F*_o_^2^) + (0.0685*P*)^2^ + 1.4008*P*] where *P* = (*F*_o_^2^ + 2*F*_c_^2^)/3
4208 reflections	(Δ/σ)_max_ < 0.001
325 parameters	Δρ_max_ = 0.73 e Å^−^^3^
0 restraints	Δρ_min_ = −0.89 e Å^−^^3^

### Special details

**Table:**

Geometry. All e.s.d.'s (except the e.s.d. in the dihedral angle between two l.s. planes) are estimated using the full covariance matrix. The cell e.s.d.'s are taken into account individually in the estimation of e.s.d.'s in distances, angles and torsion angles; correlations between e.s.d.'s in cell parameters are only used when they are defined by crystal symmetry. An approximate (isotropic) treatment of cell e.s.d.'s is used for estimating e.s.d.'s involving l.s. planes.
Refinement. Refinement of *F*^2^ against ALL reflections. The weighted *R*-factor *wR* and goodness of fit *S* are based on *F*^2^, conventional *R*-factors *R* are based on *F*, with *F* set to zero for negative *F*^2^. The threshold expression of *F*^2^ > σ(*F*^2^) is used only for calculating *R*-factors(gt) *etc*. and is not relevant to the choice of reflections for refinement. *R*-factors based on *F*^2^ are statistically about twice as large as those based on *F*, and *R*- factors based on ALL data will be even larger.

### Fractional atomic coordinates and isotropic or equivalent isotropic displacement parameters (Å^2^)

**Table:**

	*x*	*y*	*z*	*U*_iso_*/*U*_eq_	
C1	−0.5208 (4)	0.5466 (3)	0.1998 (2)	0.0413 (7)	
C2	−0.4799 (4)	0.6959 (3)	0.0737 (2)	0.0431 (7)	
C3	−0.5346 (5)	0.8171 (3)	0.0384 (3)	0.0557 (8)	
H3	−0.6217	0.8758	0.0718	0.067*	
C4	−0.4574 (5)	0.8492 (3)	−0.0477 (3)	0.0614 (10)	
H4	−0.4933	0.9306	−0.0724	0.074*	
C5	−0.3270 (5)	0.7624 (3)	−0.0982 (3)	0.0573 (9)	
H5	−0.2765	0.7858	−0.1562	0.069*	
C6	−0.2729 (4)	0.6421 (3)	−0.0623 (2)	0.0472 (7)	
H6	−0.1853	0.5839	−0.0959	0.057*	
C7	−0.3495 (4)	0.6070 (3)	0.0248 (2)	0.0382 (6)	
C8	−0.3013 (3)	0.4847 (2)	0.0697 (2)	0.0354 (6)	
C9	−0.1360 (4)	0.2793 (2)	0.0413 (2)	0.0366 (6)	
C10	0.0238 (5)	0.2595 (3)	−0.1213 (2)	0.0539 (8)	
H10A	0.0962	0.1924	−0.1587	0.081*	
H10B	0.0864	0.3110	−0.1046	0.081*	
H10C	−0.0723	0.3063	−0.1623	0.081*	
C11	−0.2074 (4)	0.2403 (2)	0.1280 (2)	0.0376 (6)	
C12	−0.3170 (3)	0.3268 (2)	0.2033 (2)	0.0353 (6)	
H12	−0.4176	0.3036	0.2181	0.042*	
C13	−0.3764 (3)	0.4543 (2)	0.1542 (2)	0.0356 (6)	
C14	−0.2271 (3)	0.3199 (2)	0.30355 (19)	0.0339 (6)	
C15	−0.0778 (3)	0.3561 (3)	0.3073 (2)	0.0368 (6)	
C16	−0.0029 (3)	0.3416 (2)	0.4100 (2)	0.0353 (6)	
C17	0.1418 (3)	0.3715 (3)	0.4272 (2)	0.0397 (6)	
H17	0.1959	0.3994	0.3731	0.048*	
C18	0.2030 (4)	0.3593 (3)	0.5248 (2)	0.0433 (7)	
C19	0.1236 (4)	0.3189 (3)	0.6067 (2)	0.0561 (8)	
H19	0.1665	0.3122	0.6723	0.067*	
C20	−0.0170 (5)	0.2890 (4)	0.5912 (2)	0.0574 (9)	
H20	−0.0704	0.2613	0.6457	0.069*	
C21	−0.0794 (4)	0.3003 (3)	0.4929 (2)	0.0419 (7)	
C22	−0.2851 (4)	0.2778 (3)	0.3880 (2)	0.0430 (7)	
H22	−0.3792	0.2537	0.3815	0.052*	
C23	−0.3248 (6)	0.0682 (4)	0.6398 (4)	0.0809 (12)	
H23	−0.3592	0.1565	0.6229	0.097*	
N1	−0.1828 (3)	0.1178 (2)	0.14793 (19)	0.0454 (6)	
N2	−0.0351 (3)	0.2123 (2)	−0.02750 (18)	0.0451 (6)	
H2	−0.0009	0.1341	−0.0163	0.054*	
O1	−0.2193 (3)	0.2669 (2)	0.48229 (15)	0.0523 (6)	
O2	−0.0185 (3)	0.3960 (2)	0.23230 (15)	0.0562 (6)	
O3	−0.6066 (3)	0.5286 (2)	0.26914 (18)	0.0568 (6)	
O4	−0.5634 (3)	0.66546 (19)	0.15818 (17)	0.0503 (5)	
O5	−0.1722 (2)	0.40090 (17)	0.01840 (14)	0.0398 (5)	
O6	−0.0894 (4)	0.0396 (2)	0.08874 (18)	0.0655 (7)	
O7	−0.2521 (3)	0.0856 (2)	0.22354 (17)	0.0560 (6)	
Cl1	0.38122 (10)	0.39733 (9)	0.54961 (7)	0.0587 (3)	
Cl2	−0.4964 (2)	0.03955 (18)	0.70060 (11)	0.1241 (6)	
Cl3	−0.1553 (2)	0.02863 (16)	0.72379 (17)	0.1416 (7)	
Cl4	−0.2684 (3)	−0.00705 (16)	0.52616 (15)	0.1326 (6)	

### Atomic displacement parameters (Å^2^)

**Table:**

	*U*^11^	*U*^22^	*U*^33^	*U*^12^	*U*^13^	*U*^23^
C1	0.0386 (15)	0.0466 (17)	0.0392 (16)	−0.0147 (13)	−0.0040 (13)	−0.0031 (13)
C2	0.0481 (17)	0.0430 (16)	0.0404 (16)	−0.0180 (13)	−0.0097 (13)	−0.0012 (13)
C3	0.064 (2)	0.0419 (17)	0.060 (2)	−0.0148 (15)	−0.0122 (17)	−0.0039 (15)
C4	0.085 (3)	0.0420 (18)	0.061 (2)	−0.0267 (18)	−0.020 (2)	0.0082 (16)
C5	0.079 (2)	0.053 (2)	0.0471 (18)	−0.0340 (18)	−0.0067 (17)	0.0062 (15)
C6	0.0574 (19)	0.0501 (18)	0.0400 (16)	−0.0261 (15)	−0.0042 (14)	−0.0002 (13)
C7	0.0446 (16)	0.0422 (15)	0.0331 (14)	−0.0210 (13)	−0.0075 (12)	−0.0025 (12)
C8	0.0381 (14)	0.0401 (15)	0.0313 (14)	−0.0168 (12)	−0.0034 (11)	−0.0061 (11)
C9	0.0428 (15)	0.0372 (15)	0.0310 (14)	−0.0145 (12)	−0.0019 (12)	−0.0045 (11)
C10	0.062 (2)	0.065 (2)	0.0367 (16)	−0.0250 (17)	0.0116 (15)	−0.0102 (15)
C11	0.0458 (16)	0.0386 (15)	0.0302 (14)	−0.0163 (12)	−0.0013 (12)	−0.0037 (11)
C12	0.0365 (14)	0.0426 (15)	0.0305 (14)	−0.0184 (12)	0.0007 (11)	−0.0027 (11)
C13	0.0368 (14)	0.0418 (15)	0.0313 (14)	−0.0167 (12)	−0.0033 (11)	−0.0050 (11)
C14	0.0375 (14)	0.0355 (14)	0.0292 (13)	−0.0130 (11)	0.0035 (11)	−0.0038 (11)
C15	0.0370 (14)	0.0421 (15)	0.0312 (14)	−0.0138 (12)	0.0050 (11)	−0.0024 (11)
C16	0.0344 (14)	0.0395 (14)	0.0299 (13)	−0.0102 (11)	0.0023 (11)	−0.0017 (11)
C17	0.0350 (14)	0.0467 (16)	0.0361 (15)	−0.0128 (12)	0.0056 (12)	−0.0031 (12)
C18	0.0346 (15)	0.0508 (17)	0.0432 (16)	−0.0115 (13)	−0.0014 (12)	−0.0096 (13)
C19	0.060 (2)	0.078 (2)	0.0334 (16)	−0.0274 (18)	−0.0082 (15)	0.0017 (15)
C20	0.065 (2)	0.086 (2)	0.0299 (16)	−0.0390 (19)	−0.0015 (15)	0.0069 (15)
C21	0.0418 (16)	0.0542 (17)	0.0327 (15)	−0.0208 (14)	0.0005 (12)	0.0008 (12)
C22	0.0453 (16)	0.0580 (18)	0.0332 (15)	−0.0276 (14)	0.0001 (12)	−0.0011 (13)
C23	0.076 (3)	0.074 (3)	0.089 (3)	−0.026 (2)	−0.010 (2)	0.026 (2)
N1	0.0586 (16)	0.0408 (14)	0.0381 (13)	−0.0187 (12)	0.0001 (12)	−0.0028 (11)
N2	0.0546 (15)	0.0425 (13)	0.0366 (13)	−0.0144 (12)	0.0062 (11)	−0.0074 (11)
O1	0.0572 (13)	0.0834 (16)	0.0298 (10)	−0.0432 (12)	−0.0001 (9)	0.0079 (10)
O2	0.0555 (13)	0.0952 (18)	0.0306 (11)	−0.0442 (13)	0.0035 (9)	0.0075 (11)
O3	0.0478 (13)	0.0641 (14)	0.0536 (14)	−0.0142 (11)	0.0137 (11)	−0.0030 (11)
O4	0.0484 (12)	0.0450 (12)	0.0520 (13)	−0.0091 (10)	0.0032 (10)	−0.0036 (10)
O5	0.0486 (11)	0.0392 (10)	0.0329 (10)	−0.0168 (9)	0.0070 (9)	−0.0046 (8)
O6	0.0964 (19)	0.0393 (12)	0.0545 (14)	−0.0152 (12)	0.0152 (13)	−0.0105 (11)
O7	0.0748 (16)	0.0496 (13)	0.0484 (13)	−0.0290 (12)	0.0069 (11)	0.0030 (10)
Cl1	0.0402 (4)	0.0789 (6)	0.0602 (5)	−0.0223 (4)	−0.0049 (4)	−0.0152 (4)
Cl2	0.1055 (10)	0.1842 (16)	0.0994 (10)	−0.0799 (11)	−0.0120 (8)	0.0481 (10)
Cl3	0.1019 (11)	0.1299 (13)	0.1951 (18)	−0.0466 (10)	−0.0691 (12)	0.0503 (12)
Cl4	0.1560 (15)	0.1120 (11)	0.1358 (14)	−0.0537 (11)	0.0268 (12)	−0.0203 (10)

### Geometric parameters (Å, º)

**Table:**

C1—O3	1.194 (4)	C12—C14	1.518 (4)
C1—O4	1.385 (4)	C12—H12	0.9800
C1—C13	1.453 (4)	C14—C22	1.328 (4)
C2—C3	1.379 (4)	C14—C15	1.454 (4)
C2—O4	1.383 (4)	C15—O2	1.225 (3)
C2—C7	1.391 (4)	C15—C16	1.477 (4)
C3—C4	1.378 (5)	C16—C21	1.387 (4)
C3—H3	0.9300	C16—C17	1.399 (4)
C4—C5	1.389 (5)	C17—C18	1.373 (4)
C4—H4	0.9300	C17—H17	0.9300
C5—C6	1.372 (4)	C18—C19	1.388 (4)
C5—H5	0.9300	C18—Cl1	1.740 (3)
C6—C7	1.400 (4)	C19—C20	1.362 (5)
C6—H6	0.9300	C19—H19	0.9300
C7—C8	1.433 (4)	C20—C21	1.387 (4)
C8—C13	1.344 (4)	C20—H20	0.9300
C8—O5	1.372 (3)	C21—O1	1.369 (3)
C9—N2	1.311 (4)	C22—O1	1.350 (3)
C9—O5	1.358 (3)	C22—H22	0.9300
C9—C11	1.392 (4)	C23—Cl3	1.737 (5)
C10—N2	1.457 (4)	C23—Cl4	1.744 (5)
C10—H10A	0.9600	C23—Cl2	1.743 (5)
C10—H10B	0.9600	C23—H23	0.9800
C10—H10C	0.9600	N1—O7	1.236 (3)
C11—N1	1.378 (4)	N1—O6	1.266 (3)
C11—C12	1.504 (4)	N2—H2	0.8600
C12—C13	1.502 (4)		
			
O3—C1—O4	117.3 (3)	C1—C13—C12	118.3 (2)
O3—C1—C13	125.8 (3)	C22—C14—C15	120.2 (2)
O4—C1—C13	116.9 (3)	C22—C14—C12	118.9 (2)
C3—C2—O4	117.4 (3)	C15—C14—C12	120.8 (2)
C3—C2—C7	121.5 (3)	O2—C15—C14	123.3 (2)
O4—C2—C7	121.2 (3)	O2—C15—C16	122.4 (3)
C4—C3—C2	118.5 (3)	C14—C15—C16	114.3 (2)
C4—C3—H3	120.7	C21—C16—C17	118.4 (2)
C2—C3—H3	120.7	C21—C16—C15	119.8 (2)
C3—C4—C5	121.3 (3)	C17—C16—C15	121.8 (2)
C3—C4—H4	119.4	C18—C17—C16	119.2 (3)
C5—C4—H4	119.4	C18—C17—H17	120.4
C6—C5—C4	119.9 (3)	C16—C17—H17	120.4
C6—C5—H5	120.1	C17—C18—C19	121.4 (3)
C4—C5—H5	120.1	C17—C18—Cl1	120.6 (2)
C5—C6—C7	120.0 (3)	C19—C18—Cl1	118.0 (2)
C5—C6—H6	120.0	C20—C19—C18	120.1 (3)
C7—C6—H6	120.0	C20—C19—H19	120.0
C2—C7—C6	118.9 (3)	C18—C19—H19	120.0
C2—C7—C8	116.5 (3)	C19—C20—C21	119.0 (3)
C6—C7—C8	124.6 (3)	C19—C20—H20	120.5
C13—C8—O5	122.9 (2)	C21—C20—H20	120.5
C13—C8—C7	123.1 (3)	O1—C21—C20	116.0 (3)
O5—C8—C7	114.0 (2)	O1—C21—C16	122.1 (2)
N2—C9—O5	111.8 (2)	C20—C21—C16	121.9 (3)
N2—C9—C11	128.3 (3)	C14—C22—O1	125.6 (3)
O5—C9—C11	119.9 (2)	C14—C22—H22	117.2
N2—C10—H10A	109.5	O1—C22—H22	117.2
N2—C10—H10B	109.5	Cl3—C23—Cl4	112.0 (3)
H10A—C10—H10B	109.5	Cl3—C23—Cl2	109.4 (2)
N2—C10—H10C	109.5	Cl4—C23—Cl2	111.7 (3)
H10A—C10—H10C	109.5	Cl3—C23—H23	107.9
H10B—C10—H10C	109.5	Cl4—C23—H23	107.9
N1—C11—C9	120.6 (2)	Cl2—C23—H23	107.9
N1—C11—C12	116.3 (2)	O7—N1—O6	120.7 (2)
C9—C11—C12	123.1 (2)	O7—N1—C11	119.6 (2)
C13—C12—C11	108.8 (2)	O6—N1—C11	119.7 (2)
C13—C12—C14	111.9 (2)	C9—N2—C10	125.4 (3)
C11—C12—C14	112.5 (2)	C9—N2—H2	117.3
C13—C12—H12	107.8	C10—N2—H2	117.3
C11—C12—H12	107.8	C22—O1—C21	117.9 (2)
C14—C12—H12	107.8	C2—O4—C1	122.5 (2)
C8—C13—C1	119.5 (3)	C9—O5—C8	119.7 (2)
C8—C13—C12	122.1 (2)		
			
O4—C2—C3—C4	−177.9 (3)	C12—C14—C15—O2	1.5 (4)
C7—C2—C3—C4	0.2 (5)	C22—C14—C15—C16	−0.1 (4)
C2—C3—C4—C5	−0.2 (5)	C12—C14—C15—C16	−178.8 (2)
C3—C4—C5—C6	−0.1 (5)	O2—C15—C16—C21	177.6 (3)
C4—C5—C6—C7	0.2 (5)	C14—C15—C16—C21	−2.1 (4)
C3—C2—C7—C6	−0.1 (4)	O2—C15—C16—C17	−0.7 (4)
O4—C2—C7—C6	178.0 (2)	C14—C15—C16—C17	179.6 (2)
C3—C2—C7—C8	178.8 (3)	C21—C16—C17—C18	−0.1 (4)
O4—C2—C7—C8	−3.2 (4)	C15—C16—C17—C18	178.2 (3)
C5—C6—C7—C2	−0.1 (4)	C16—C17—C18—C19	−0.5 (4)
C5—C6—C7—C8	−178.9 (3)	C16—C17—C18—Cl1	−179.2 (2)
C2—C7—C8—C13	0.4 (4)	C17—C18—C19—C20	0.7 (5)
C6—C7—C8—C13	179.2 (3)	Cl1—C18—C19—C20	179.5 (3)
C2—C7—C8—O5	−180.0 (2)	C18—C19—C20—C21	−0.4 (6)
C6—C7—C8—O5	−1.2 (4)	C19—C20—C21—O1	179.0 (3)
N2—C9—C11—N1	−3.6 (5)	C19—C20—C21—C16	−0.2 (5)
O5—C9—C11—N1	174.8 (2)	C17—C16—C21—O1	−178.7 (3)
N2—C9—C11—C12	177.8 (3)	C15—C16—C21—O1	3.0 (4)
O5—C9—C11—C12	−3.7 (4)	C17—C16—C21—C20	0.4 (5)
N1—C11—C12—C13	−161.8 (2)	C15—C16—C21—C20	−177.9 (3)
C9—C11—C12—C13	16.9 (4)	C15—C14—C22—O1	1.6 (5)
N1—C11—C12—C14	73.6 (3)	C12—C14—C22—O1	−179.7 (3)
C9—C11—C12—C14	−107.8 (3)	C9—C11—N1—O7	−177.5 (3)
O5—C8—C13—C1	−175.4 (2)	C12—C11—N1—O7	1.1 (4)
C7—C8—C13—C1	4.2 (4)	C9—C11—N1—O6	2.6 (4)
O5—C8—C13—C12	4.0 (4)	C12—C11—N1—O6	−178.7 (3)
C7—C8—C13—C12	−176.5 (2)	O5—C9—N2—C10	−4.0 (4)
O3—C1—C13—C8	173.3 (3)	C11—C9—N2—C10	174.6 (3)
O4—C1—C13—C8	−6.0 (4)	C14—C22—O1—C21	−0.9 (5)
O3—C1—C13—C12	−6.0 (4)	C20—C21—O1—C22	179.3 (3)
O4—C1—C13—C12	174.6 (2)	C16—C21—O1—C22	−1.5 (4)
C11—C12—C13—C8	−16.9 (3)	C3—C2—O4—C1	179.3 (3)
C14—C12—C13—C8	108.1 (3)	C7—C2—O4—C1	1.2 (4)
C11—C12—C13—C1	162.5 (2)	O3—C1—O4—C2	−176.0 (3)
C14—C12—C13—C1	−72.6 (3)	C13—C1—O4—C2	3.4 (4)
C13—C12—C14—C22	123.2 (3)	N2—C9—O5—C8	167.0 (2)
C11—C12—C14—C22	−113.9 (3)	C11—C9—O5—C8	−11.7 (4)
C13—C12—C14—C15	−58.0 (3)	C13—C8—O5—C9	11.8 (4)
C11—C12—C14—C15	64.9 (3)	C7—C8—O5—C9	−167.8 (2)
C22—C14—C15—O2	−179.7 (3)		

### Hydrogen-bond geometry (Å, º)

Cg4 is the centroid of the C2–C7 ring.

**Table:**

*D*—H···*A*	*D*—H	H···*A*	*D*···*A*	*D*—H···*A*
N2—H2···O6	0.86	1.00	2.604 (3)	127
N2—H2···O6^i^	0.86	2.17	2.910 (3)	144
C6—H6···O2^ii^	0.96	2.49	3.186 (4)	132
C10—H10*B*···*Cg*4^ii^	0.98	2.98	3.719 (4)	134

Symmetry codes: (i) −*x*, −*y*, −*z*; (ii) −*x*, −*y*+1, −*z*.
