# Supplementary figures and images for: Crystal structure of 4-(6-chloro-4-oxo-4H-chromen-3-yl)-2-methyl­amino-3-nitro-4H,5H-pyrano[3,2-c]chromen-5-one chloro­form monosolvate
Source: Acta Crystallogr E Crystallogr Commun. 2015 Jun 27;71(Pt 7):o512–3. doi: 10.1107/S2056989015011810 (PMC4518942; doi:10.1107/S2056989015011810)

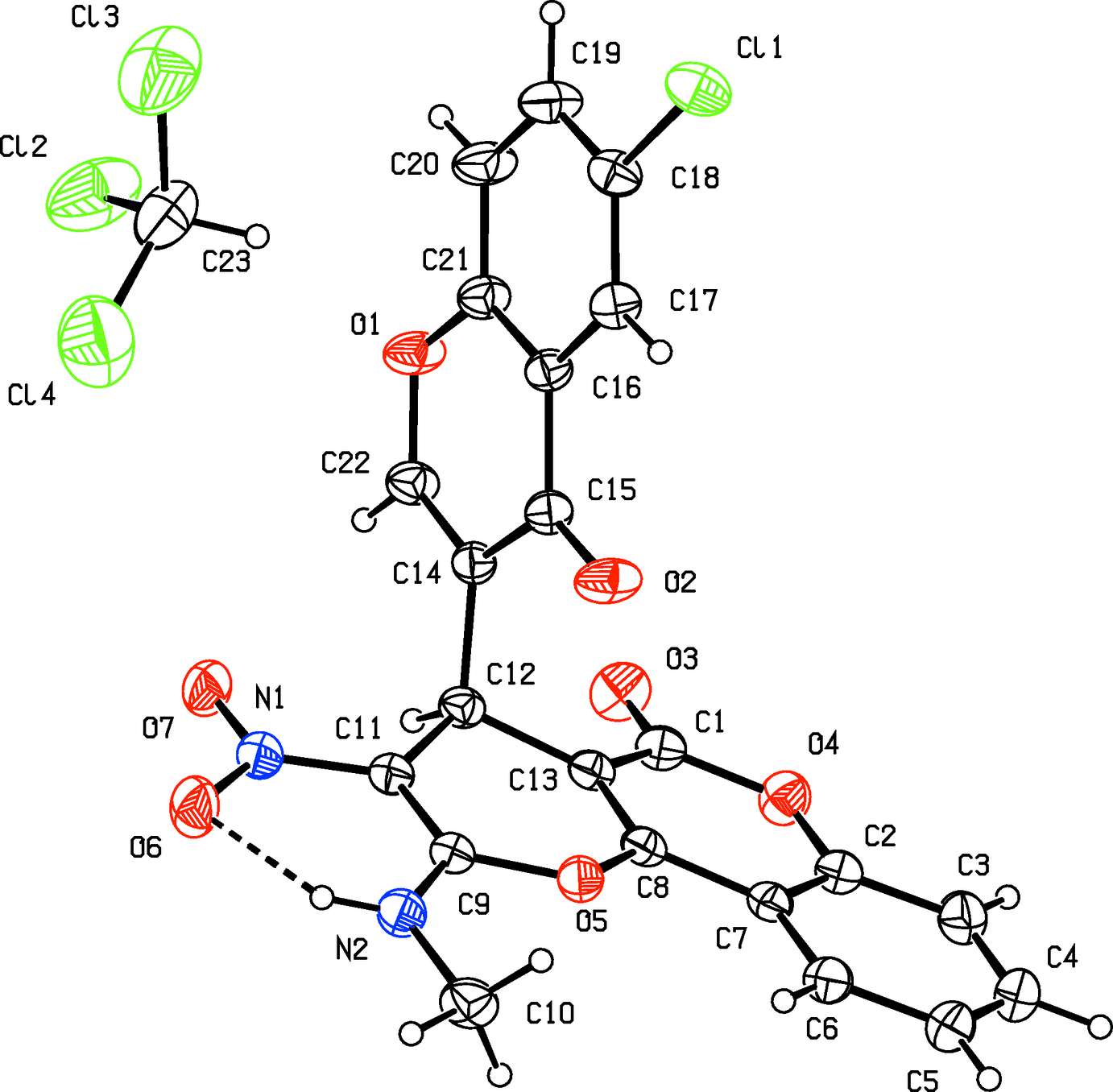

Supplement: Supplementary file 4 [file e-71-0o512-fig1.tif]

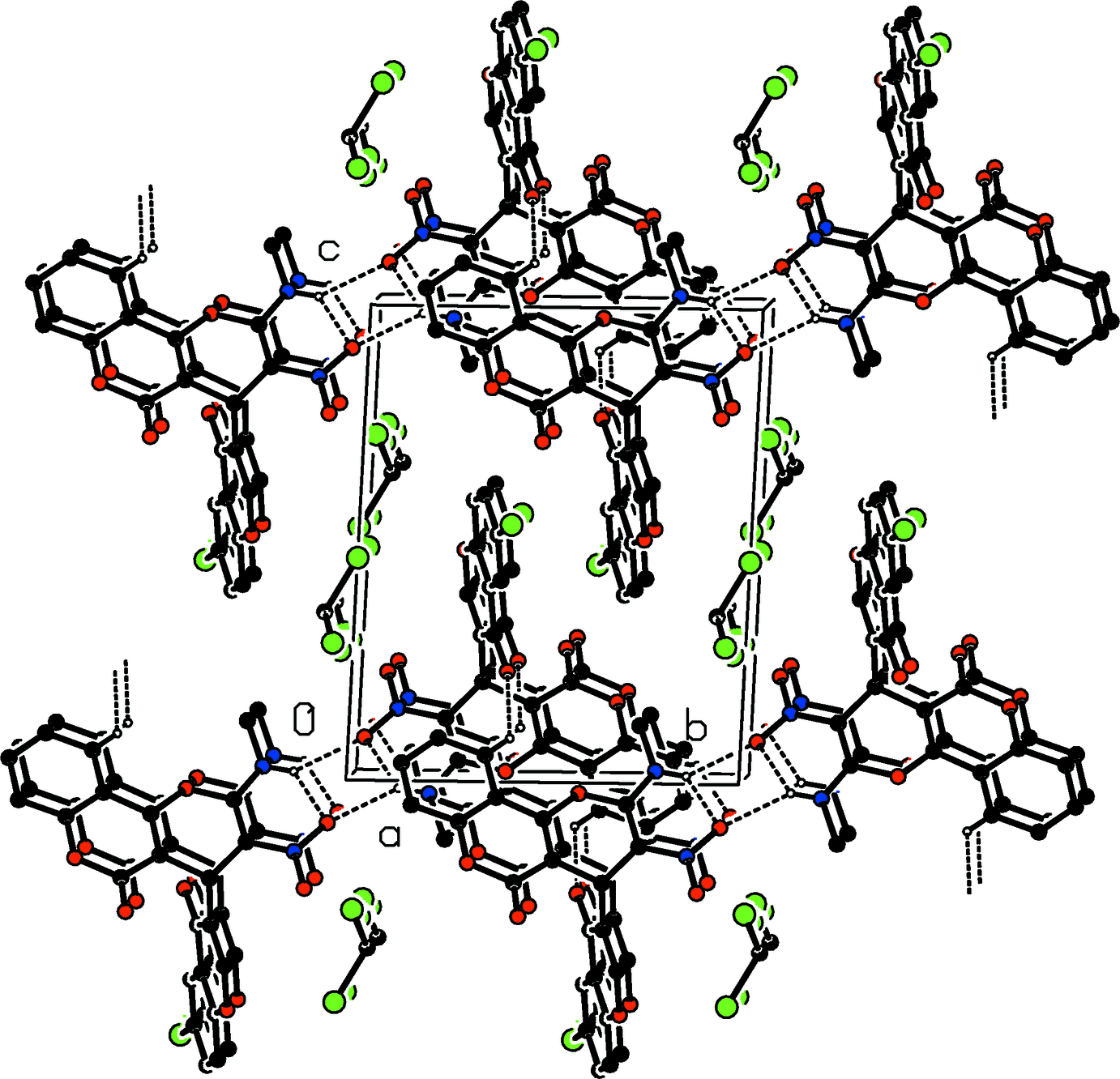

Supplement: Supplementary file 5 [file e-71-0o512-fig2.tif]
